# Occurrence and diagnostic of intermittent shedding of *Staphylococcus aureus* in bovine mammary infection

**DOI:** 10.3389/fvets.2025.1523698

**Published:** 2025-02-11

**Authors:** Lena Mues, Nicole Kemper, Julia Anna Blumenberg

**Affiliations:** ^1^Institute of Animal Hygiene, Animal Welfare and Livestock Ethology, University of Veterinary Medicine Hannover, Hannover, Germany; ^2^Faculty of Agricultural and Nutritional Science, Institute of Animal Breeding and Husbandry, University of Kiel, Kiel, Germany

**Keywords:** *Staphylococcus aureus*, bovine mastitis, intermittent shedding, *S. aureus* diagnostic, *S. aureus* mastitis

## Abstract

Bovine mastitis is a major problem with huge economic losses in dairy farming worldwide. One of the most common pathogens is *Staphylococcus aureus*, which is highly contagious and often spread during milking. A sanitation of a dairy herd can be challenging particularly in terms of diagnostics, because of intermittent shedding of *Staphylococcus aureus* in milk. The observation of intermittent shedding of *Staphylococcus aureus* in longitudinal studies and applied detection methods were reviewed in this study. Categorization of detection methods is used to describe the basic influence of intermittent shedding on sensitivity of diagnostic of each category. The laboratory diagnostic methods evaluated have a wide range regarding the detection limit (40 cfu/mL−10^6^ cfu/mL). A low detection limit is essential for the detection of even chronically infected cows with intermittent shedding of the pathogen. The literature overview shows that only a few studies (*n* = 6) examined occurrence of intermittent shedding of *Staphylococcus aureus* in milk at cow level. A detection-free period of ≤ 0.5–1 d was only observed in 3 studies.

## Introduction

Bovine mastitis is the most common disease in dairy herds and leads to major economic losses in dairy farms (1). *Staphylococcus (S.) aureus* is one of the most frequently occurring mastitis pathogens, causes huge costs (2) and can lead to chronical intramammary infections. In a recent prevalence study from Lower Saxony, Germany, *S. aureus* was detected in bulk milk in 18.3% of the investigated dairy herds (3). Diagnosis is particularly difficult as the pathogen is shed cyclically, and only small quantities of the pathogen are excreted at times (4, 5). Intermittent shedding patterns and the resulting diagnostic challenges have also been investigated in other mastitis pathogens such as *Prototheca* spp. and *Mycoplasma* spp. (6, 7). *S. aureus* has different pathogenic properties to circumvent the immune response of the host (8), e.g., the ability to form a biofilm is suggested to be a major factor (9), although biofilm formation *in vivo* has rarely been investigated (10). While pathogen quantities of 55,000 cfu/mL are excreted in severe cases of mastitis (11), the excretion of *S. aureus* in milk is partially below the detection limit of standard laboratory diagnostics (12–14). False negative results due to such intermittent shedding can prevent the successful sanitation of a dairy, therefore research should prioritize on early detection to control the disease through treatment and management (15). The aim of this mini review is to obtain an overview on the occurrence of intermittent *S. aureus*-shedding in milk and to evaluate the sampling strategies and diagnostic possibilities.

## Sampling strategies

The excretion of *S. aureus* in milk can differ greatly from day to day and even from two infected quarters of the same cow (5). Sears et al. (4) were able to show that the detection rate of *S. aureus* could be increased from 75 % to 94 % and respectively 98 % following a second and third sample of the quarter. The time of milk sampling and handling also was found to be relevant: the likelihood of detecting subclinical *S. aureus-*infection is higher in fresh milk samples taken before milking than in samples taken after milking (16). Villanueva et al. (17) suspected that freezing milk samples could destroy bacterial cell aggregates, which would improve the sensitivity of BC. Such a positive effect could not be confirmed in comparison with fresh pre-milking samples in a study by Godden et al. (16). The freezing of milk samples seems to have different effects on pathogens, whereby no effect could be confirmed especially for *S. aureus* (18). A study with centrifuged milk samples showed that cultures from the sediment of quarter milk samples can increase the number of positive results by up to 145.5 % (19). Furthermore, Mahmmod et al. (20) demonstrated that pre-sampling procedures (cleaning, disinfecting and discarding first milked streaks) significantly reduced the likelihood of false-positive *S. aureus* results by eliminating colonies from the skin and teat canal.

Hence, for detecting pathogens in milk different sample types can be used. Quarter milk sample (QMS) are the standard method used for the detection of intramammary infections (IMI) (21). Maisano et al. (22) suggested that the serial sampling of composite milk samples is an alternative to a few QMS. To assess the infection status of a herd, bulk milk samples could be analyzed for the prevalence of *S. aureus* (23, 24). Britten (13) recommended the targeted use of selective media in general for composite and bulk milk samples to improve sensitivity. However, these results do not allow any statement to be made about the prevalence of the pathogen in individual cows.

## Detection methods

### Microbiological cultivation

In mastitis diagnostics bacterial culture (BC) is standard for detection of bovine IMI. For *S. aureus*, different selective media have been reported. The use of Baird Parker agar (BP), Vogel-Johnson (VJ), Champman agar (CHAP), CHROMagar *S. aureus* (CHROM) and chromID *S. aureus* (SAID) were compared by Graber et al. (27). BP, VJ, and CHAP are used for diagnostic of bovine originated isolates whereas the CHROM and SAID have been evaluated for use of *S. aureus*-isolates from human origin (27). However, in this study the authors concluded that the specificity of the different selective media is unsatisfactory, mainly because of the similar reactions and occurrence of non-aureus staphylococci (27). In food industry BP is used for enumeration of gram-positive staphylococci, i.e. *S. aureus* (28). Baird and Lee (29) rated BP as standard medium for enumeration of *S. aureus*. Artursson et al. (30) investigated eight methods for the isolation of *S. aureus* from bovine milk samples varying different culture volumes, enrichment, incubation and freezing methods, as well as sedimentation and use of Mastistrip cassette (SVA, Uppsala, Sweden). They concluded that pre-incubation of milk without additives at 37°C for 18 hours increased the number of positive udder quarters by 50%. Middleton et al. (31) stated that in routine mastitis diagnostics with BC, the standard application volume is 10 μl and therefore the minimum detection limit is 100 cfu/mL (see Table 1). Walker et al. (32) demonstrated that the inoculum size of 0.1 mL was found to be the most accurate size for detecting a *S. aureus*-infection. In addition, the growth of only one colony forming unit (cfu) was classified as sufficient for a positive result (32, 33). However, it was found that the sensitivity of a single sample can be up to 90 % if all cultures (including mixed cultures) are considered positive for *S. aureus* at a threshold of 1 cfu/0,01mL (34). Furthermore, mixed infections and low pathogen excretion can complicate the diagnosis of *S. aureus* using BC (35). More evidence could also be obtained for mixed cultures through standardized thresholds and definitions as described by Dohoo et al. (34). The suitable use of culture improvement methods can significantly increase the sensitivity of detection of mastitis organisms in milk (13). The definition of an infection or a positive finding in the BC ranges from ≥1cfu/0.1 mL (32), ≥2 cfu/0,1/mL (12), ≥3 cfu/0.1 mL (36) to ≥1 cfu/0.01 mL (5, 33). These different definitions have a major influence on studies on the occurrence of intermittent shedding. Consequently, the authors had different opinions whether there was cyclical intermittent shedding, in which cultural detection is not possible (4, 37), or rather only low shedding, which is detectable with a larger inoculum (32).

**Table 1:** Detection methods and reported detection limits in the diagnosis of *Staphylococcus aureus* from milk.

**No**.	**Method**	**Detection limit (cfu/mL)**	**Reference**
1	BC	100	(34)
2	BC	100	(31)
3	Serology	100	(40)
4	Serology	10,000	(43)
5	qPCR	100	(47)
6	qPCR	40	(52)
7	Isothermal amplification	900	(54)
8	Isothermal amplification	100	(53)
9	Isothermal amplification	2,000	(55)
10	Isothermal amplification	500	(56)
11	Mass spectrometry	≥10^6^	(64)
12	Mass spectrometry	100	(65)

### Serology

Several rapid immunological tests for the detection of *S. aureus* in milk have been developed. ELISA rapid test for the detection of antigen-specific IgG was investigated by Yang and Laven (38) and El- Rashidy et al. (39) for the detection of *S. aureus* in milk samples. The sensitivity of the tests was found to be 94 % (31) and 86 % (32), but the detection limit is not reported. Another Biosensing method was used to detect *S. aureus* from milk by binding to the *Fc* fragment of human IgG on bio-functionalized beads and detected using antibodies labeled by fluorescence markers. Here, the detection limit in milk was 100 cfu/mL (40). In another ELISA-based technique, the enzyme thermostable nuclease (TNase) produced by *S. aureus* was detected. In this method, a combination of immunomagnetic separation and ELISA (IMS-ELISA) was developed and tested on composite milk samples from 444 cows. The detection limit was approximately 10^5^ *S. aureus* per mL milk (41). The sensitivity was considered to be limited, and it was stated that TNase is not specific for *S. aureus* as also other coagulase-positive staphylococci (*S. intermedius* and *S. hyicus*), as well as some coagulase-negative staphylococci produce TNase (41, 42). A further rapid test based on immunology, an immunochromatographic strip test (ICS) was developed and reported with a detection limit of 10^4^ cfu/mL (43). In all 3 studies (40, 41, 43) that determined a detection limit used spiked milk samples. Antibody tests are not dependent on the shedding pattern of the bacterium and in addition fast and relatively cheap (44). Many serological detection methods for *S. aureus* are rapid tests, but with a detection limit that might be unsatisfactory for the detection of subclinical infections (43). Furthermore, it must be noted that there might be a discrepancy between antibodies and the actual amount of pathogens excreted (45).

### Molecular diagnostic

There are many different approaches in molecular diagnostics for example thermocycle methods as conventional PCR or qPCR as well as isothermal methods are used. Many protocols apply for direct DNA-extraction from milk samples (46–48). The selected protocol and performance of DNA extractions has a major influence on the result, for example gram-positive bacteria such as *S. aureus*, extraction is challenging, as these bacteria often remain in the cream fraction (49). In conventional PCR each protocol defines a specific number of amplification cycles before the determination the qPCR runs a determination after each cycle. Hence, the result is not only negative or positive but allows also for graduation of positive results (50). Cederlöf et al. (51) using Ct-value cut offs and proposed that low PCR-Ct-values could be defined as “truly/strongly infection” whereas high Ct-values could be defined as “*S. aureus*- positive cow.” In studies with qPCR, detection limits of 40 cfu/mL (52) and 100 cfu/mL (47) were determined. In opposite to conventional PCR and qPCR, isothermal methods amplify in constant temperature and, hence, do not need to apply a thermocycler. The detection limits for isothermal method (see Table 1) range from 1 × 10^2^ cfu/mL to 2 × 10^3^ cfu/mL in milk (53–56). Studer et al. (36) compared the sensitivity of the qPCR protocol of Graber et al. (57) with the sensitivity of classical BC in the examination of chronically infected *S. aureus* quarter milk samples. They summarized that the sensitivity was 92.9% for qPCR and 21.4% for BC, with a low pathogen shedding rate, and concluded that one sample per quarter examined with qPCR was sufficient to obtain a definitive result. Nevertheless, the sensitivity of PCR diagnostics is also dependent on the shedding pattern and requires investments in equipment that usually exceeds those of BC (38). Furthermore, a PCR assay detects the DNA of viable and non-viable bacteria, whereas BC only detects viable bacteria (58). Consequently, PCR could detect dead DNA from infections that have already subsided (13). Such cases have hardly been investigated and the relevance of PCR-positive and culture-negative results should be focused on future research (13).

### Mass spectrometry

In the early years of the use of mass spectrometry in mastitis research, it was based on single protein analysis (59–61). Hettinga et al. (62) attempted to link the analysis of volatile bacterial metabolites to certain mastitis pathogens. Barreiro et al. (63) started to use mass spectrometry to detect complete mastitis pathogens and considered the species identification of mastitis isolates using matrix-assisted laser desorption/ionization time of flight mass spectrometry (MALDI TOF MS) for faster identification than by biochemical methods. Barreiro et al. (64) developed a protocol to investigate the opportunity of direct detection of bacteria in milk without previous BC using MALDI TOF MS. Experimentally contaminated skim milk identified adequate (score ≥2) *S. aureus* with concentrations ≥10^6^ cfu/mL (see Table 1). Sauerbrey (65) demonstrated a detection limit of 10^2^ cfu/mL with spiked skim milk in a validation test (see Table 1), but bacteria identification from raw milk samples was not possible without doing BC beforehand. The use of MALDI TOF MS for identification of mastitis pathogen is time consuming with previous BC. The approaches of Barreiro et al. and Sauerbrey (64, 65) to identify the pathogens directly from milk are promising. However, this current detection limit is not satisfactory as only clinical mastitis with extremely high bacterial shedding can be detected.

In the future, the use of AI might also be an option to identify bacteria genera and species based on colony morphology, although there is still limited literature available. Garcia et al. (25) compared an AI-based plate reading application with MALDI-TOF MS on clinical mastitis-causing pathogens and had difficulties to differentiate non-aureus Staphylococci from *S. aureus*. In addition, the colonies within *Staphylococcus* spp. are quite similar in morphology, which makes differentiation and thus diagnosis quite difficult (26). Currently this is not an option for the detection of intermittent bacterial species such as *S. aureus*, as the AI cannot recognize anything unless there is any growth on the plate.

## Enrichment

In general, enrichment processes are applied before microbial cultures and initiated in specific sampling situations (21). Keefe (14) assumed that the level of *S. aureus*-excretion may be below the detection limit of conventional BC and stated that enrichment media could be a helpful tool to increase sensitivity in case of low *S. aureus*-shedding. Also in human medicine for isolation of *S. aureus* from swab sample selective enrichment broths were used (66, 67). Furthermore, enrichment media for *S. aureus* were used to investigate the food safety of milk powder and compared with direct plating at selective Baird Parker medium and Hauschild pork plasma fibrinogen medium (68). In this study, the enrichment medium (Giolitti Cantoni broth with Tween 80) did not achieve better results than direct plating on Baird Parker medium but compared to direct plating on Hauschild medium it did (68). Only a few studies have investigated the potential of enrichment broths for more sensitive bovine mastitis diagnostics with different results (30, 69, 70): Thurmond et al. (70) showed in an experiment with composite milk samples that pre-enrichment of milk samples in Brain heart fusion increases the probability of isolating *S. aureus* by 1.6 times. Artursson et al. (30) could not demonstrate positive effect on the isolation of *S. aureus* with nutrient broth containing 10% horse serum in analyses of subclinical quarter milk samples. In some studies an enrichment of the milk was done before PCR to carry out more sensitive diagnostics (71–73). Further studies with different enrichment media for the detection of subclinical *S. aureus*-infections could provide further insights.

## Occurrence of intermittent shedding in raw milk

Intermittent shedding of *S. aureus* in bovine milk samples at cow level was investigated in six longitudinal studies shown in Table 2 (4, 5, 12, 33, 36, 39). Despite an extensive literature search from 1980 to 2024, only these six studies provided information on the shedding of *S. aureus* at cow level, of which five had detailed information on intermittent shedding.

**Table 2:** Comparison of longitudinal studies investigating intermittent shedding of *Staphylococcus aureus* in bovine milk samples.

**Sampling object**	**Sampling—time**	**Cows (*n*)**	**Naturally infected**	**Intermittent shed—cows (*n*)**	**Duration of non-detected shed**	**Reference**
56 QMS/19 *glands*	28 d	7	No	*-* 16 *glands*	≥0.5 d	(4)
30 QMS/cow	16 d	4	Yes	*-*	*-*	(4)
130QMS/13 *glands*	10 d	10	Yes	2^c^	1 d	(39)
132QMS^a^/22 *glands*^a^	6 d	16^a^	Yes	*-* 11 QMS negative	*-*	(12)
154 QMS/11 *glands*	14 d	10	Yes	1	1 d	(36)
397 QMS/9 *glands*	3 × 21 d	7	Yes	*-*	*-*	(5)
1,070 QMS/29 *glands*	26–44 weeks	22	Yes	2^b^	*-*	(33)

^a^only *S. aureus* positive samples/cows included; ^b^No information at the time of positive results, one cow (2 infected glands) 77% and 85% positive samples, another cow 39% positive samples. ^c^The two cows tested negative on the last day of sampling.

The six studies differ slightly in scope of sample, with 4–22 *S. aureus* infected cows being analyzed, all examined QMS using BC as detection method. Studer et al. (36) additionally made use of qPCR (see Table 2). Sears et al. (4) investigated naturally and artificially infected cows with *S. aureus* in an experimental model. In four of the longitudinal studies, the duration of sampling ranged from 6 d to 28 d with at least one sample taken daily. In a study by Walker et al. (5) samples are taken three times on 21 consecutive days during lactation. In another study (33) quarter milk samples are taken weekly over a period of 26–44 weeks. In those studies (5, 33) the bacterial genome was additionally analyzed using pulsed field electrophoresis. Consequently, it should be noted that the six studies differ greatly in terms of study design, which makes comparability difficult.

In three studies, undetectable intervals (see Table 2; “Duration of non-detected shed”) were not investigated in detail, only three studies mention undetectable intervals of ≥0.5 d or 1 d (4, 36, 39). Walker et al. (5) determined that 97.5% of the samples were positive. Unfortunately, the duration of non-detected shed and the number of those cows was not reported. Similar to the study by Buelow et al. (12) in which 11 negative QMS are mentioned, but the number of cows and time interval were not discussed.

Walker et al. (33) compared the results of their study with those of Sears et al. (4) and concluded that naturally infected mammary glands excrete *S. aureus* in a more consistent pattern than experimentally infected mammary glands. In addition it was found that *S. aureus* strains have different affinities for the mammary gland, which is why the strain selection might achieve different results (33, 74). However, Sears et al. and Walker et al. (4, 33) agreed on the definition of different types of shedding, so-called low shedding pattern (≤ 10 cfu/0.01 mL) and high shedding pattern (≥20 cfu/0.01 mL). Furthermore, Walker et al. and Studer et al. (33, 36) both observed sinusoidal shedding pattern, i.e., an alternation between low and high *S. aureus* shedding over time. Studer et al. (36) suggested that this is a result of a synchronized process between the pathogen and the immune system. However, Walker et al. (33) noted that the duration and amplitude of each pattern varied, so that no consistent pattern was observed between or within cows. In a few longitudinal studies transmission of *S. aureus* was examined on herd level and focusing on the transmission of *S. aureus* examining the different genotypes (75, 76). Sommerhäuser (75) concluded that the persistence of the pathogen in the udder tissue is more likely in herd sub-suspensions than a temporary cure and subsequent reinfection by the same *S. aureus-*type. On the other hand Wente et al. (77) investigated recurrent clinical mastitis and stated that *S. aureus* showed the highest recurrence rate (27 % of all *S. aureus* cases).

## Conclusion

In summary, the occurrence of intermittent shedding of *Staphylococcus aureus* and the need for sensitive diagnostics is to be investigated in further longitudinal studies. Particular attention should be paid in future research to the duration of undetectable shedding. As this duration was reported as very short in the studies by Sears et al. and Studer et al. (4, 36), close monitoring should also be carried out in future research. Furthermore, whole genome sequencing of the isolates could provide further insights into microevolution in the host in order to determine the persistence of the bacterium in the udder or a re-infection. It is therefore important that a consistent terminology is established to characterize IMI over time (78). Furthermore, there is no standardized definition for the diagnosis of an IMI with *S. aureus* (32). For BC a sample could already be considered positive if 1 cfu grows in an inoculum of 0.1 mL, as stated by Walker et al. (32, 33). For molecular diagnostics, the subdivision into infected and positive depending on the Ct-value, which was proposed by Cederlöf et al. (51), could be useful. Definitions need to be clarified to enhance research in the field of intermittent shedding of *S. aureus* in bovine mastitis. Regarding the detection limit, the serological detection and direct MALDI TOF MS studies require further investigations to achieve detection even with low shedding.
